# Supplementary material for: Low nitrogen availability inhibits the phosphorus starvation response in maize (Zea mays ssp. mays L.)
Source: BMC Plant Biol. 2021 Jun 5;21:259. doi: 10.1186/s12870-021-02997-5 (PMC8178920; doi:10.1186/s12870-021-02997-5)
Supplement: Supplementary file 1 — Additional file 1: Figure S1. Growth traits for plants grown under Full, LowN, LowP and LowNP. Plots show estimated coefficient and associated standard error. The significance of the treatment effect is shown as *** p < 0.001, ** p < 0.01, * p < 0.05, p < 0.1 (Kruskal-Wallis test; p-value adjusted for multiple tests). Lowercase letters indicate significant (p < 0.05) pairwise differences (Dunn test). Figure accompanies MZ66_Growth_Analysis in Supplemental File 1. [file 12870_2021_2997_MOESM1_ESM.pdf]

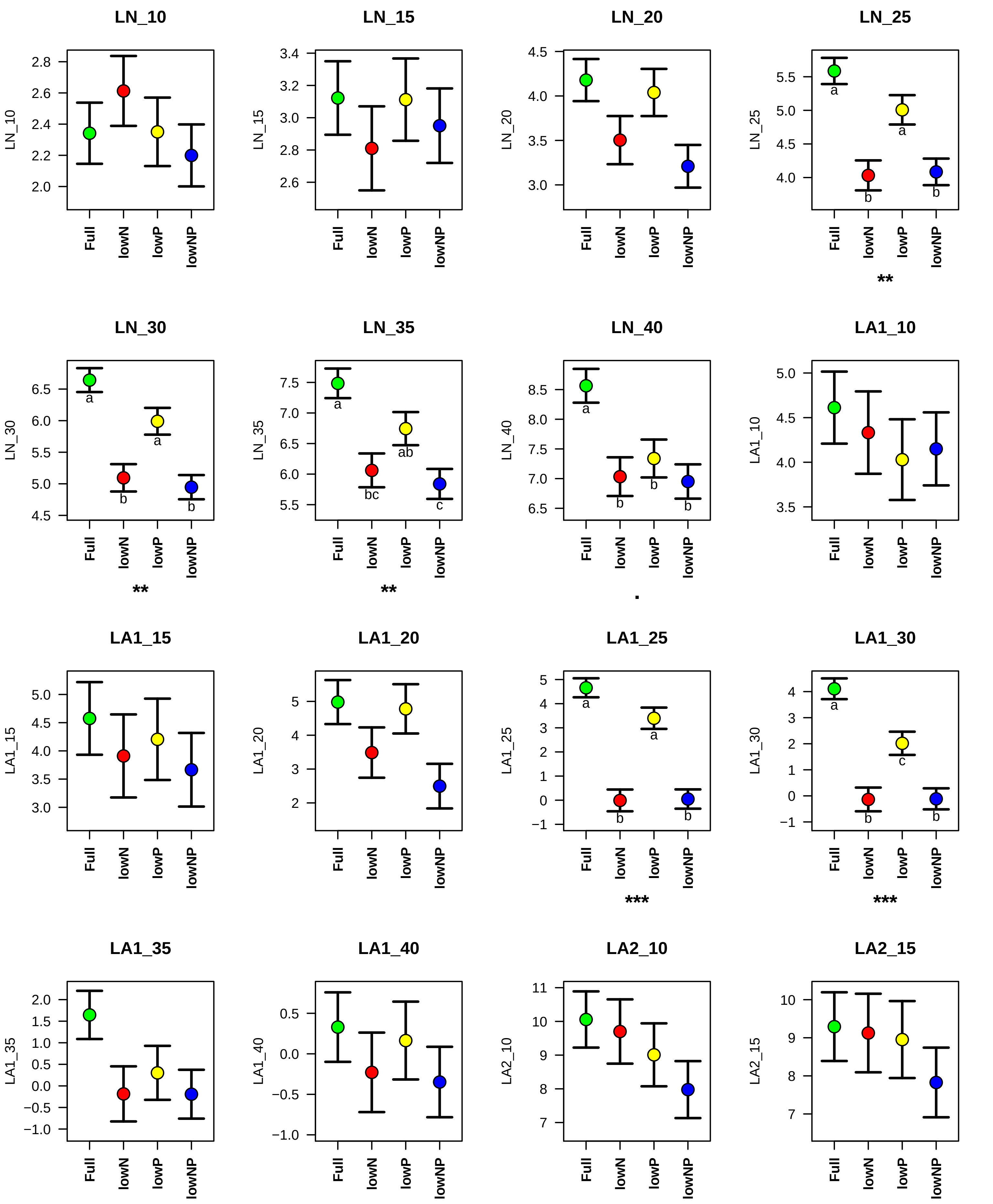
LN_10
LN_15
LN_20
LN_25
**
LN_30
**
LN_35
**
LN_40
.
LA1_10
LA1_15
LA1_20
LA1_25
***
LA1_30
***
LA1_35
LA1_40
LA2_10
LA2_15
Full
lowN
lowP
lowNP

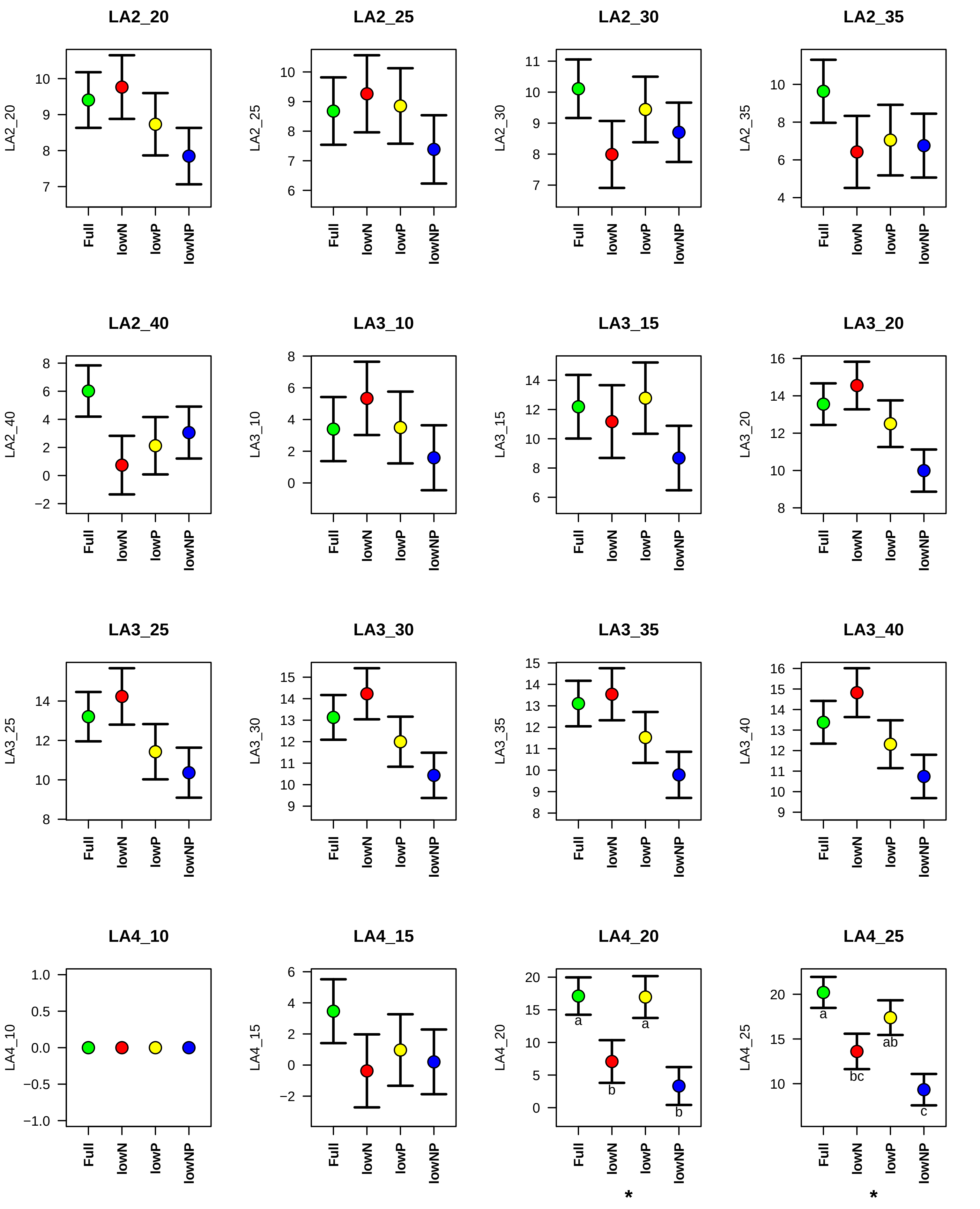
LA2_20
LA2_25
LA2_30
LA2_35
LA2_40
LA3_10
LA3_15
LA3_20
LA3_25
LA3_30
LA3_35
LA3_40
LA4_10
LA4_15
LA4_20
LA4_25
Full
lowN
lowP
lowNP
a
b
a
b
*
a
bc
ab
c
*

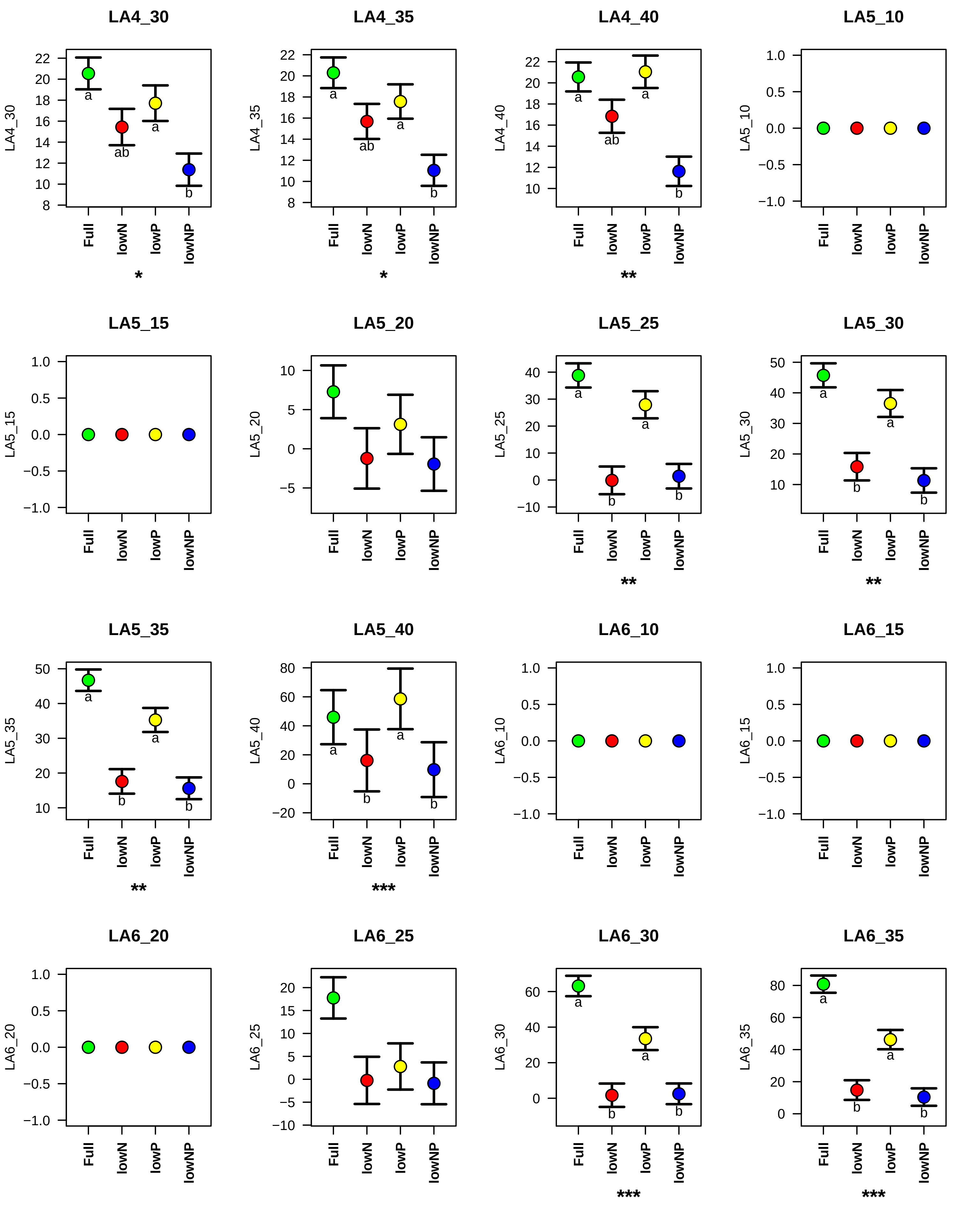
LA4_30
LA4_30
a ab a b
Full lowN lowP lowNP
*
LA4_35
LA4_35
a ab a b
Full lowN lowP lowNP
*
LA4_40
LA4_40
a ab a b
Full lowN lowP lowNP
**
LA5_10
LA5_10
Full lowN lowP lowNP
LA5_15
LA5_15
Full lowN lowP lowNP
LA5_20
LA5_20
Full lowN lowP lowNP
LA5_25
LA5_25
a b a b
Full lowN lowP lowNP
**
LA5_30
LA5_30
a b a b
Full lowN lowP lowNP
**
LA5_35
LA5_35
a b a b
Full lowN lowP lowNP
**
LA5_40
LA5_40
a b a b
Full lowN lowP lowNP
***
LA6_10
LA6_10
Full lowN lowP lowNP
LA6_15
LA6_15
Full lowN lowP lowNP
LA6_20
LA6_20
Full lowN lowP lowNP
LA6_25
LA6_25
Full lowN lowP lowNP
LA6_30
LA6_30
a b a b
Full lowN lowP lowNP
***
LA6_35
LA6_35
a b a b
Full lowN lowP lowNP
***

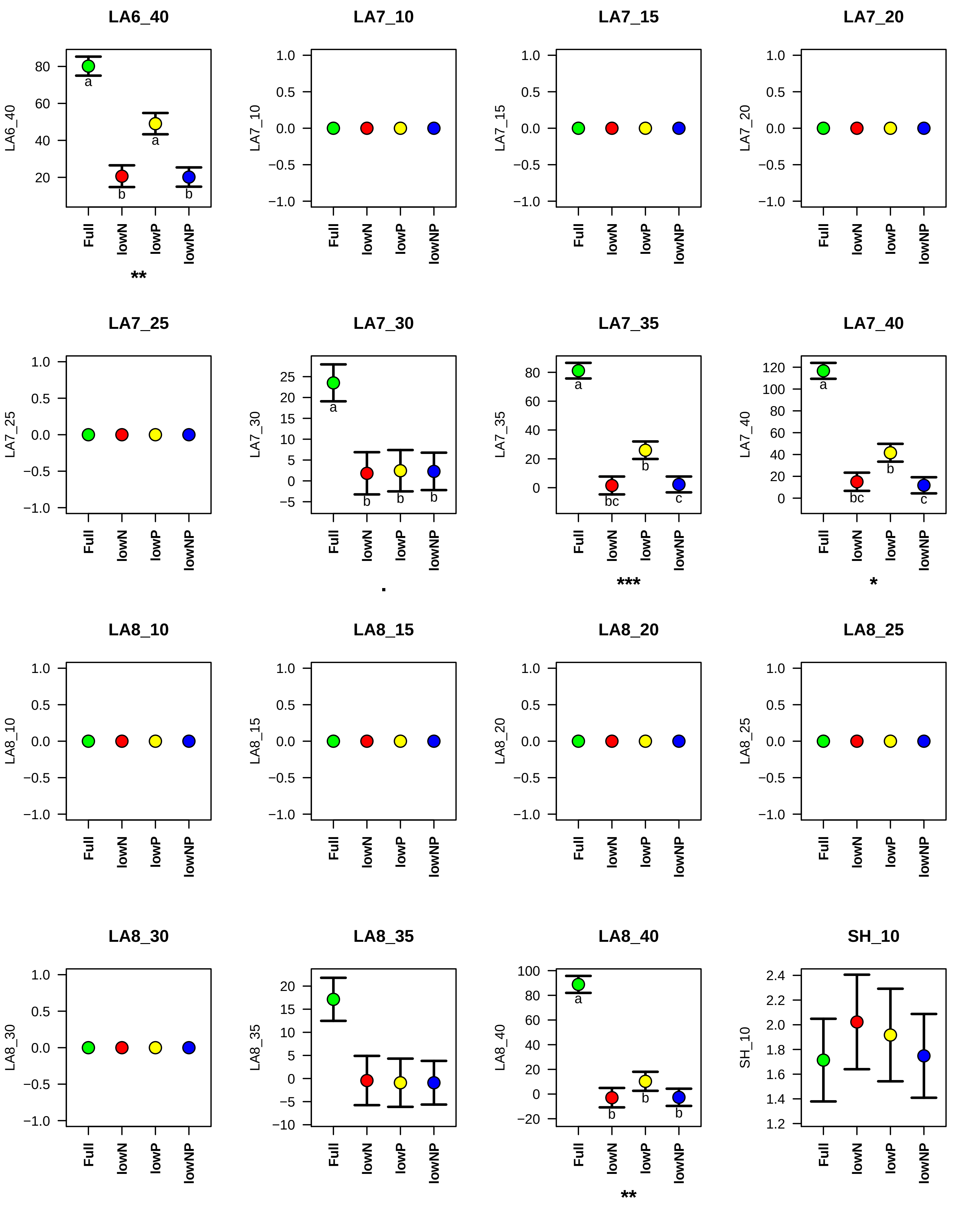
LA6_40
80
60
40
20
a
b
a
b
Full
lowN
lowP
lowNP
**
LA7_10
1.0
0.5
0.0
−0.5
−1.0
LA7_15
LA7_20
LA7_25
LA7_30
25
20
15
10
5
0
−5
a
b
b
b
.
LA7_35
bc
c
***
LA7_40
120
100
bc
c
*
LA8_10
LA8_15
LA8_20
LA8_25
LA8_30
LA8_35
−10
LA8_40
−20
**
SH_10
2.4
2.2
2.0
1.8
1.6
1.4
1.2

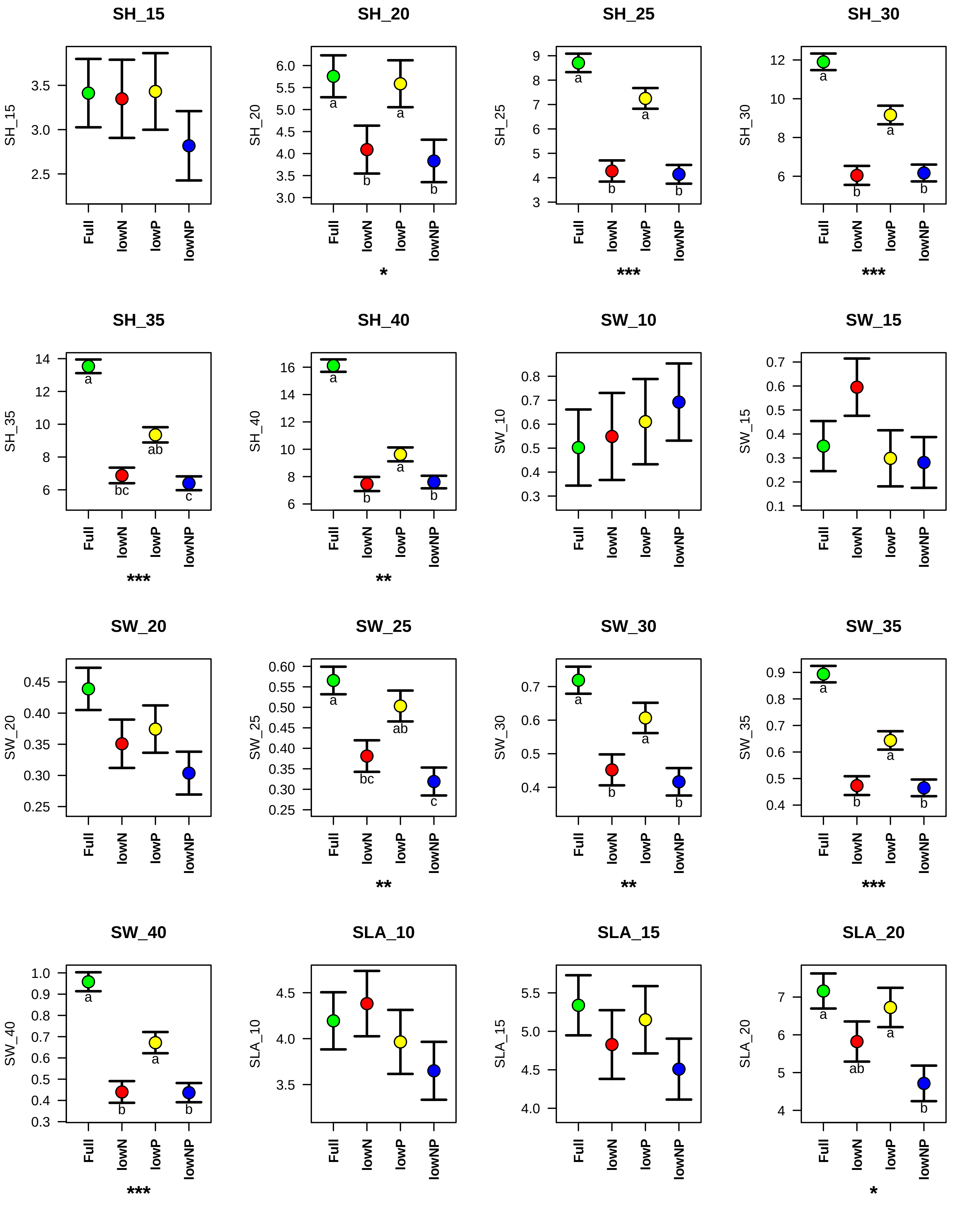
SH_15
SH_15
3.5
3.0
2.5
Full
lowN
lowP
lowNP
SH_20
SH_20
6.0
5.5
5.0
4.5
4.0
3.5
3.0
a
b
a
b
Full
lowN
lowP
lowNP
*
SH_25
SH_25
9
8
7
6
5
4
3
a
b
a
b
Full
lowN
lowP
lowNP
***
SH_30
SH_30
12
10
8
6
a
b
a
b
Full
lowN
lowP
lowNP
***
SH_35
SH_35
14
12
10
8
6
a
bc
ab
c
Full
lowN
lowP
lowNP
***
SH_40
SH_40
16
14
12
10
8
6
a
b
a
b
Full
lowN
lowP
lowNP
**
SW_10
SW_10
0.8
0.7
0.6
0.5
0.4
0.3
Full
lowN
lowP
lowNP
SW_15
SW_15
0.7
0.6
0.5
0.4
0.3
0.2
0.1
Full
lowN
lowP
lowNP
SW_20
SW_20
0.45
0.40
0.35
0.30
0.25
Full
lowN
lowP
lowNP
SW_25
SW_25
0.60
0.55
0.50
0.45
0.40
0.35
0.30
0.25
a
bc
ab
c
Full
lowN
lowP
lowNP
**
SW_30
SW_30
0.7
0.6
0.5
0.4
a
b
a
b
Full
lowN
lowP
lowNP
**
SW_35
SW_35
0.9
0.8
0.7
0.6
0.5
0.4
a
b
a
b
Full
lowN
lowP
lowNP
***
SW_40
SW_40
1.0
0.9
0.8
0.7
0.6
0.5
0.4
0.3
a
b
a
b
Full
lowN
lowP
lowNP
***
SLA_10
SLA_10
4.5
4.0
3.5
Full
lowN
lowP
lowNP
SLA_15
SLA_15
5.5
5.0
4.5
4.0
Full
lowN
lowP
lowNP
SLA_20
SLA_20
7
6
5
4
a
ab
a
b
Full
lowN
lowP
lowNP
*

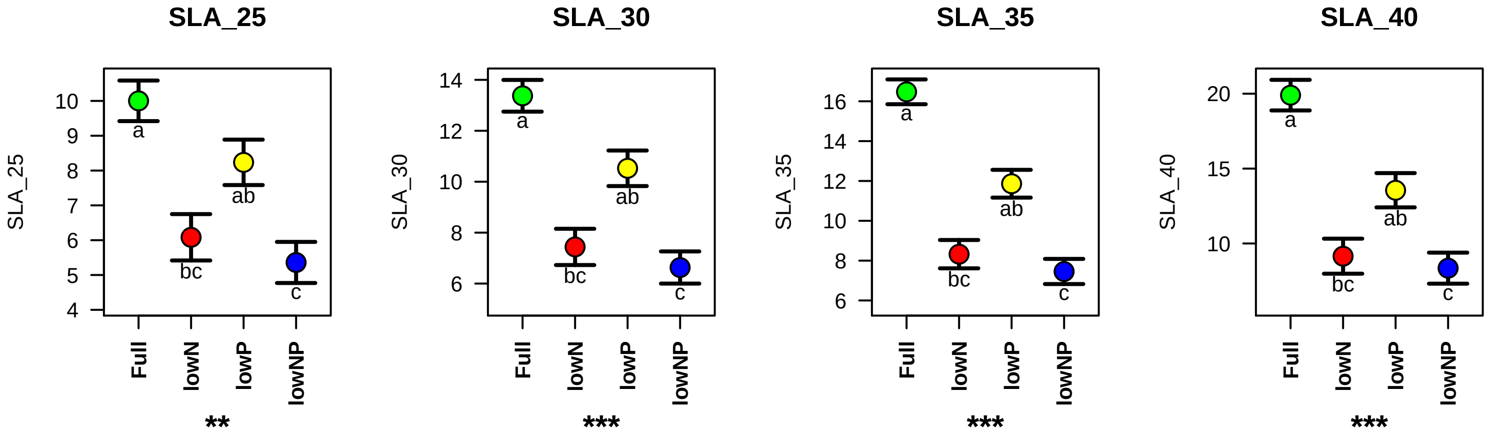

SLA_25
SLA_30
SLA_35
SLA_40
SLA_25
SLA_30
SLA_35
SLA_40
a
bc
ab
c
Full
lowN
lowP
lowNP
**
***
***
***
