# Supplementary material for: Low nitrogen availability inhibits the phosphorus starvation response in maize (Zea mays ssp. mays L.)
Source: BMC Plant Biol. 2021 Jun 5;21:259. doi: 10.1186/s12870-021-02997-5 (PMC8178920; doi:10.1186/s12870-021-02997-5)
Supplement: Supplementary file 2 — Additional file 2: Figure S2. Endpoint traits for plants grown under Full, LowN, LowP and LowNP. Plots show estimated coefficient and associated standard error. The significance of the treatment effect is shown as *** p < 0.001, ** p < 0.01, * p < 0.05, p < 0.1 (Kruskal-Wallis test; p-value adjusted for multiple tests). Lowercase letters indicate significant (p < 0.05) pairwise differences (Dunn test). Figure accompanies MZ66_Endpoint_Analysis in Supplemental File 1. [file 12870_2021_2997_MOESM2_ESM.pdf]

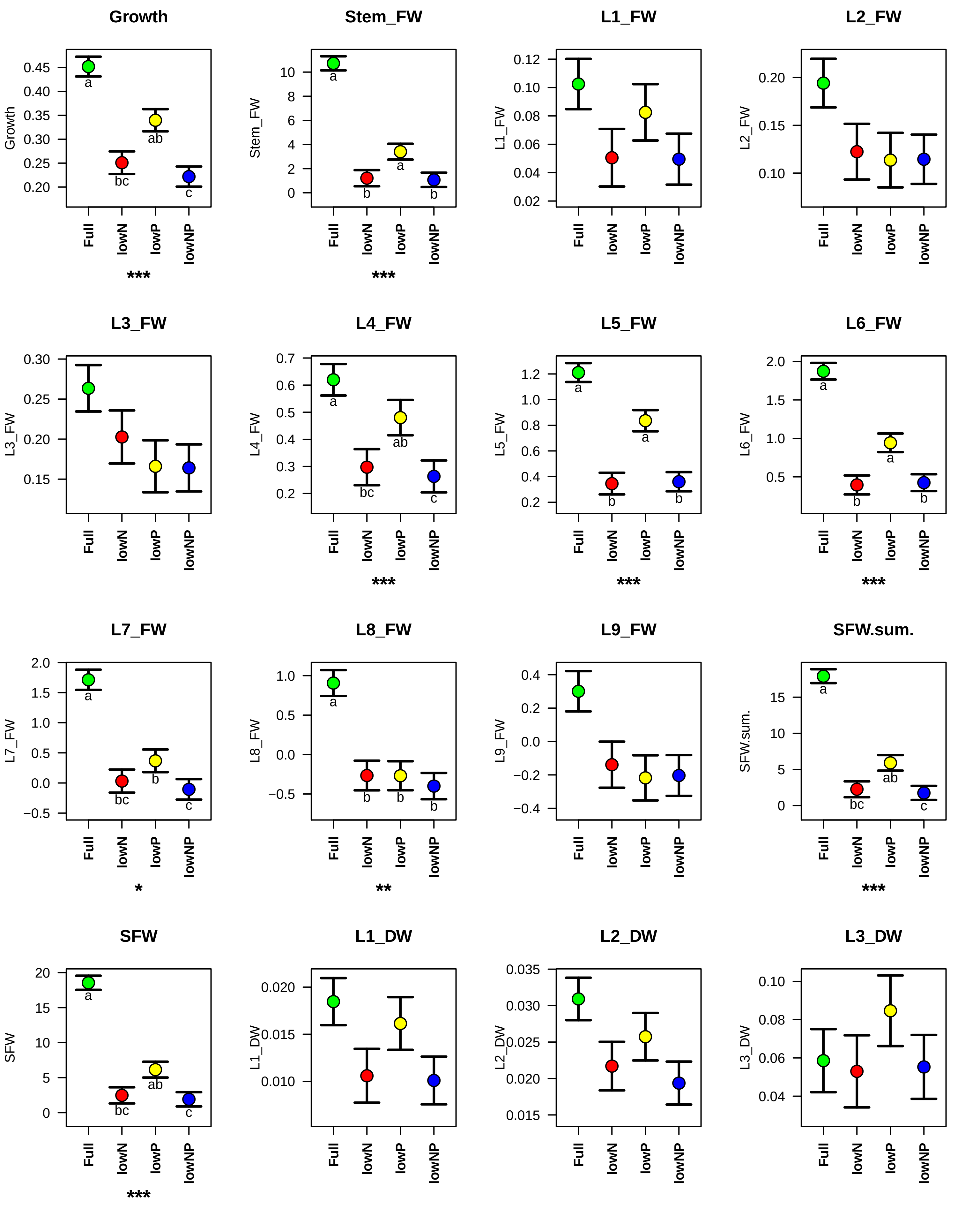
Growth
Growth
0.45
0.40
0.35
0.30
0.25
0.20
a
bc
ab
c
Full
lowN
lowP
lowNP
***
Stem_FW
Stem_FW
10
8
6
4
2
0
a
b
a
b
Full
lowN
lowP
lowNP
***
L1_FW
L1_FW
0.12
0.10
0.08
0.06
0.04
0.02
Full
lowN
lowP
lowNP
L2_FW
L2_FW
0.20
0.15
0.10
Full
lowN
lowP
lowNP
L3_FW
L3_FW
0.30
0.25
0.20
0.15
Full
lowN
lowP
lowNP
L4_FW
L4_FW
0.7
0.6
0.5
0.4
0.3
0.2
a
bc
ab
c
Full
lowN
lowP
lowNP
***
L5_FW
L5_FW
1.2
1.0
0.8
0.6
0.4
0.2
a
b
a
b
Full
lowN
lowP
lowNP
***
L6_FW
L6_FW
2.0
1.5
1.0
0.5
a
b
a
b
Full
lowN
lowP
lowNP
***
L7_FW
L7_FW
2.0
1.5
1.0
0.5
0.0
−0.5
a
bc
b
c
Full
lowN
lowP
lowNP
*
L8_FW
L8_FW
1.0
0.5
0.0
−0.5
a
b
b
b
Full
lowN
lowP
lowNP
**
L9_FW
L9_FW
0.4
0.2
0.0
−0.2
−0.4
Full
lowN
lowP
lowNP
SFW.sum.
SFW.sum.
15
10
5
0
a
bc
ab
c
Full
lowN
lowP
lowNP
***
SFW
SFW
20
15
10
5
0
a
bc
ab
c
Full
lowN
lowP
lowNP
***
L1_DW
L1_DW
0.020
0.015
0.010
Full
lowN
lowP
lowNP
L2_DW
L2_DW
0.035
0.030
0.025
0.020
0.015
Full
lowN
lowP
lowNP
L3_DW
L3_DW
0.10
0.08
0.06
0.04
Full
lowN
lowP
lowNP

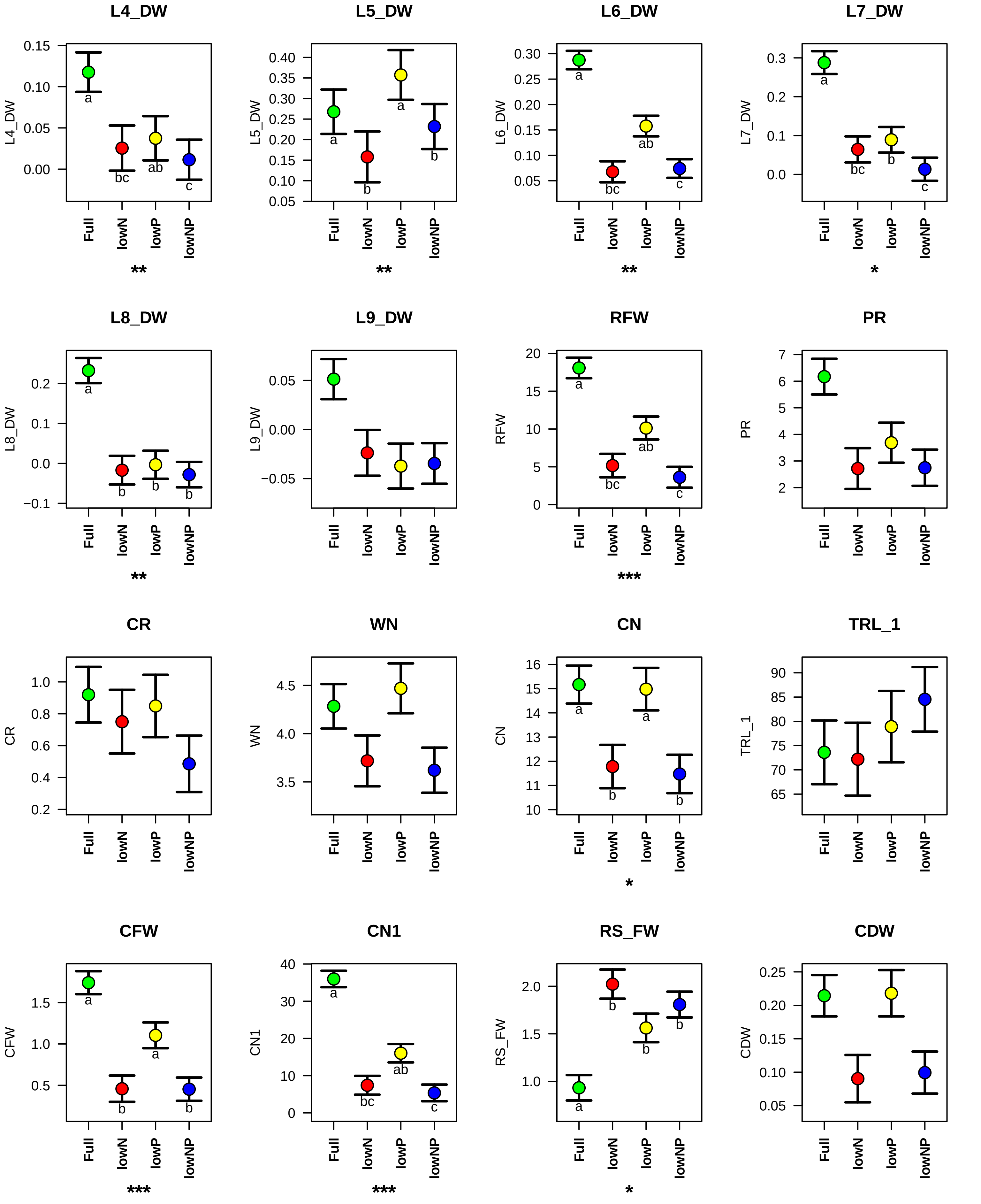
L4_DW
**
L5_DW
**
L6_DW
**
L7_DW
*
L8_DW
**
L9_DW
RFW
***
PR
CR
WN
CN
*
TRL_1
CFW
***
CN1
***
RS_FW
*
CDW
Full
lowN
lowP
lowNP
a
ab
bc
b
c

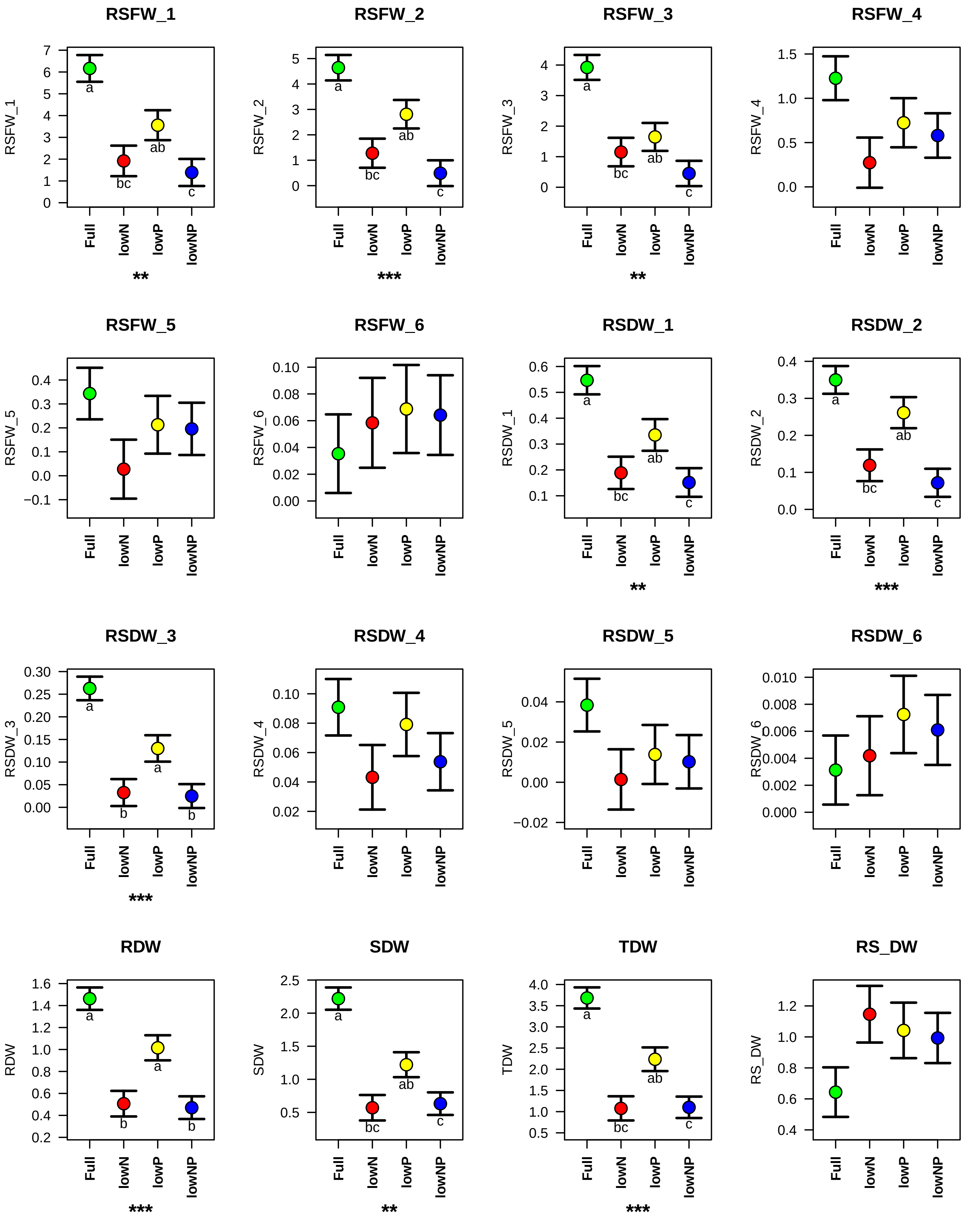
RSFW_1
RSFW_1
a bc ab c
**
RSFW_2
RSFW_2
a bc ab c
***
RSFW_3
RSFW_3
a bc ab c
**
RSFW_4
RSFW_4
RSFW_5
RSFW_5
RSFW_6
RSFW_6
RSDW_1
RSDW_1
a bc ab c
**
RSDW_2
RSDW_2
a bc ab c
***
RSDW_3
RSDW_3
a b a b
***
RSDW_4
RSDW_4
RSDW_5
RSDW_5
RSDW_6
RSDW_6
RDW
RDW
a b a b
***
SDW
SDW
a bc ab c
**
TDW
TDW
a bc ab c
***
RS_DW
RS_DW
Full
lowN
lowP
lowNP

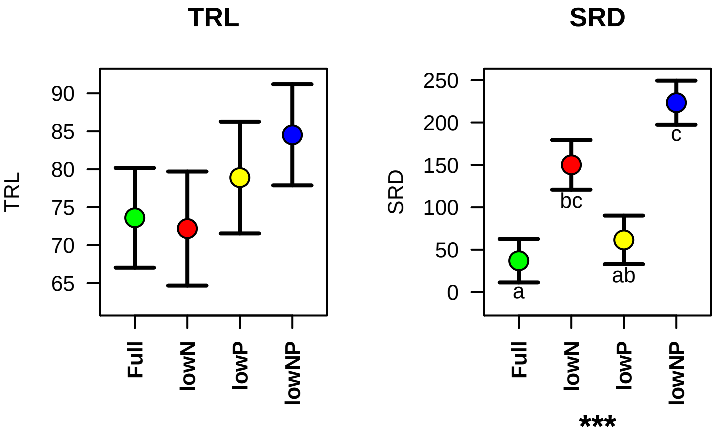

TRL
TRL
90
85
80
75
70
65
Full
lowN
lowP
lowNP
SRD
SRD
250
200
150
100
50
0
a
bc
ab
c
Full
lowN
lowP
lowNP
***
