# Supplementary figures and images for: Low nitrogen availability inhibits the phosphorus starvation response in maize (Zea mays ssp. mays L.)
Source: BMC Plant Biol. 2021 Jun 5;21:259. doi: 10.1186/s12870-021-02997-5 (PMC8178920; doi:10.1186/s12870-021-02997-5)

ARW

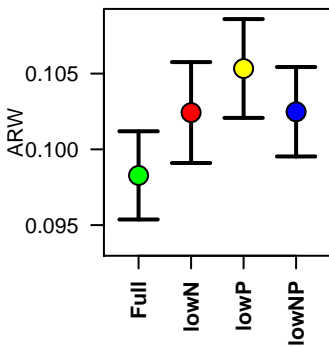

EAR

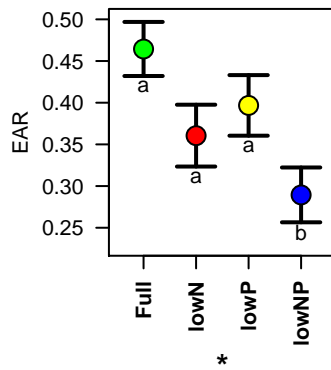

MaxNR

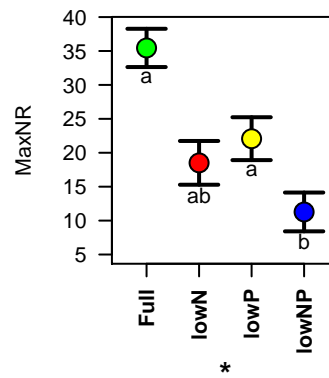

MEA

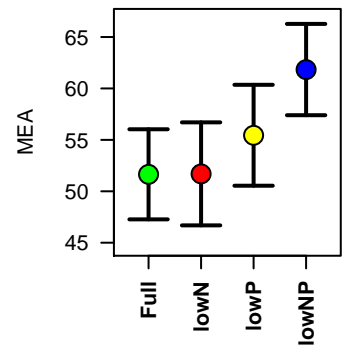

MinEA

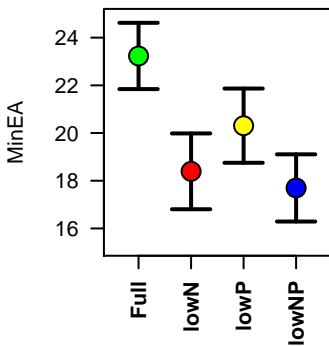

MNR

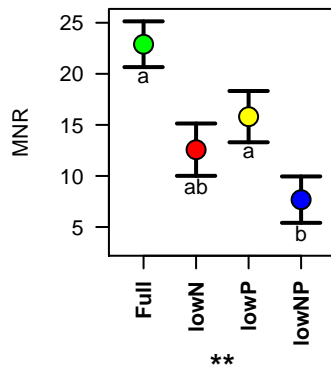

NB

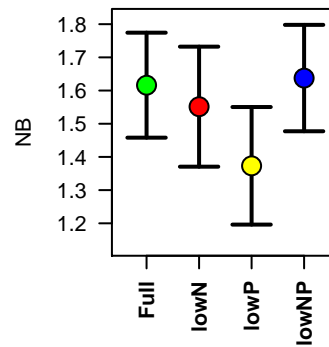

NCA

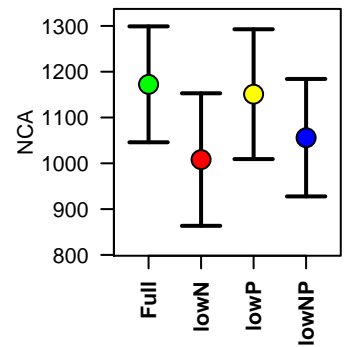

NCC

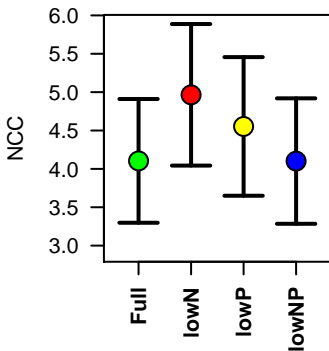

ND

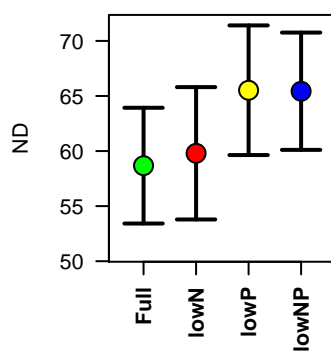

NetA

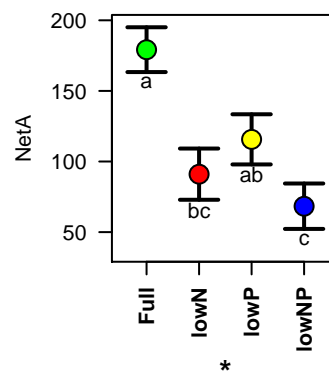

NL

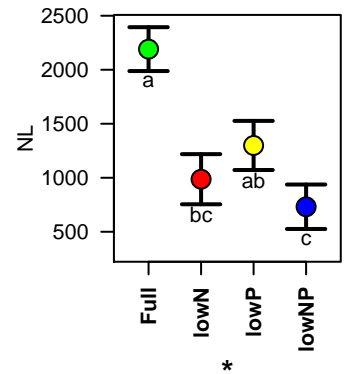

NLD

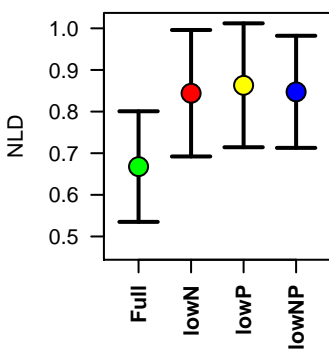

NP

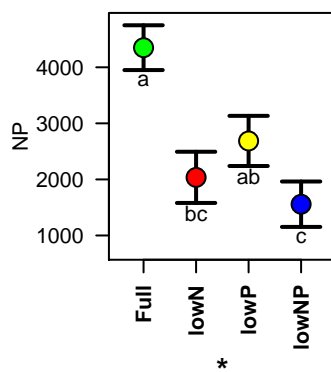

NS

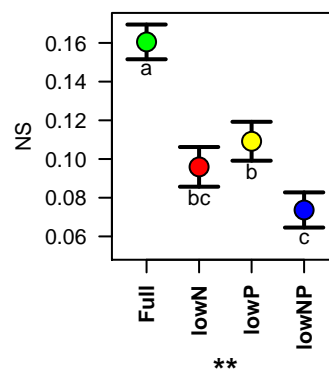

NSA

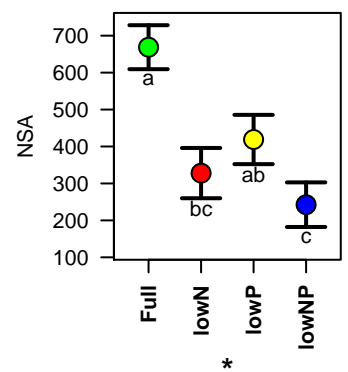

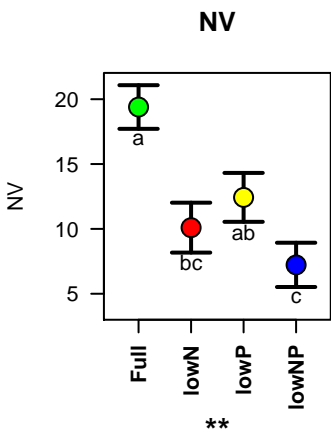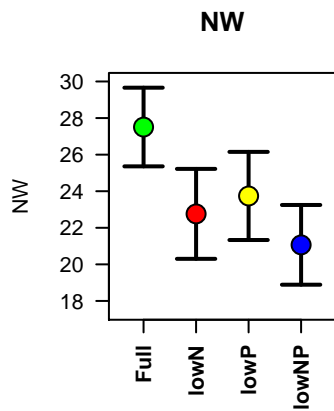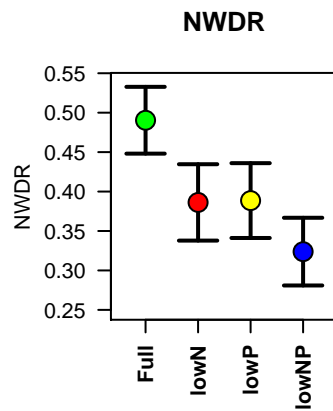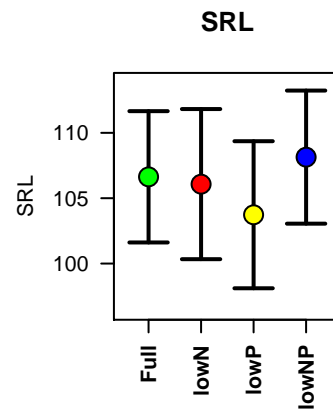

Supplement: Supplementary file 3 — Additional file 3: Figure S3. GiaRoot root features for plants grown under Full, LowN, LowP and LowNP. Plots show estimated coefficient and associated standard error. The significance of the treatment effect is shown as *** p < 0.001, ** p < 0.01, * p < 0.05, p < 0.1 (Kruskal-Wallis test; p-value adjusted for multiple tests). Lowercase letters indicate significant (p < 0.05) pairwise differences (Dunn test). Figure accompanies MZ66_Giaroots_Analysis in Supplemental File 1. [file 12870_2021_2997_MOESM3_ESM.pdf]

Al

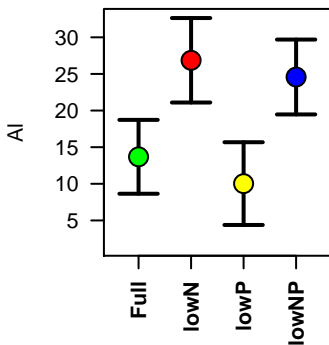

As

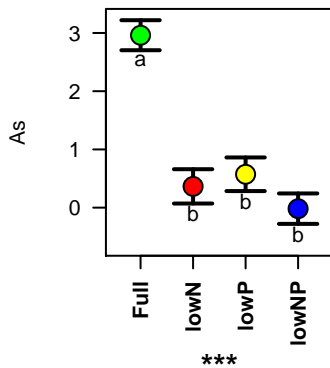

B

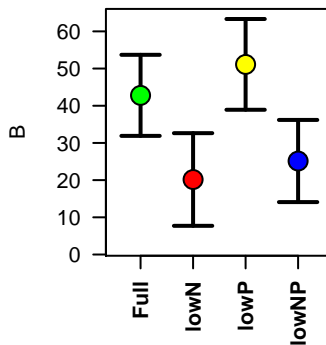

Ca

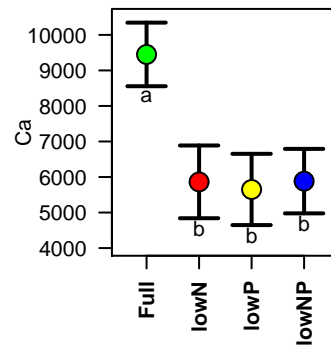

Cd

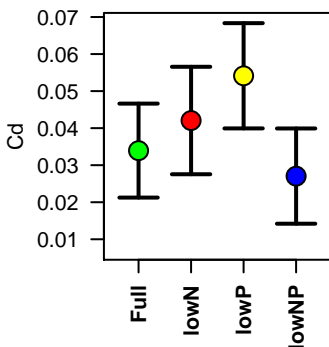

Co

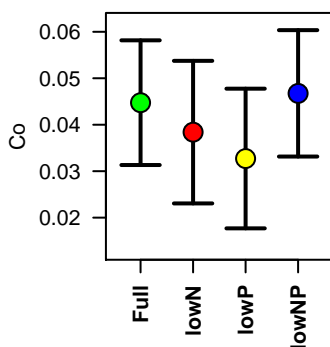

Cu

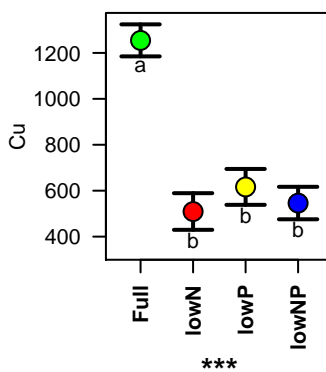

Fe

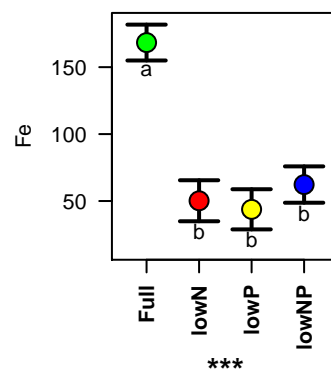

K

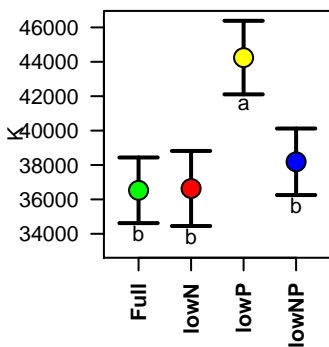

Mg

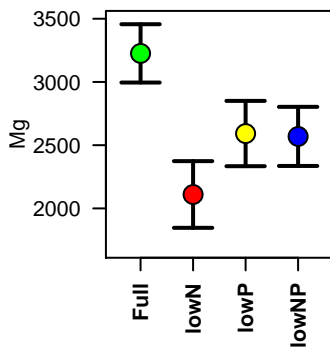

Mn

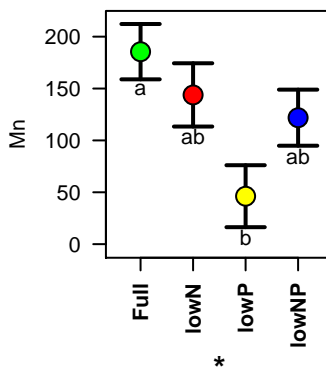

Mo

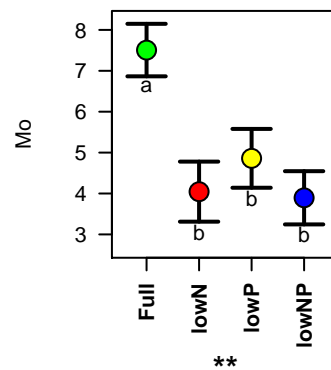

Na

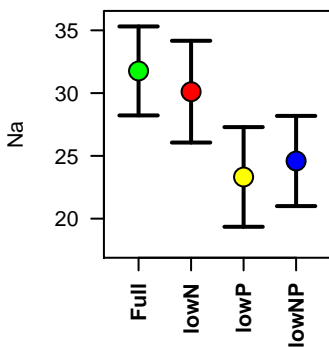

Ni

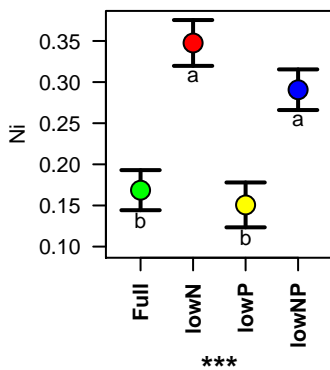

P

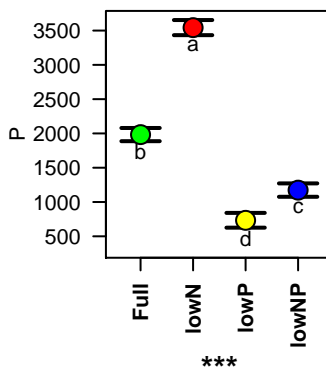

Rb

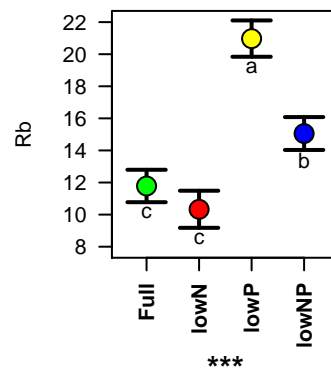

S

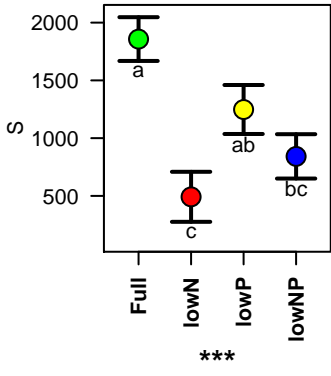

Se

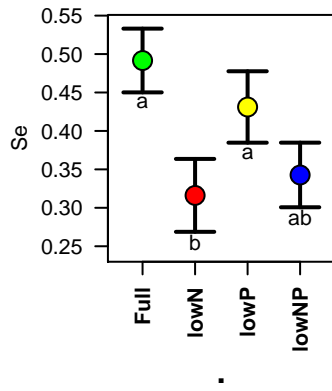

Sr

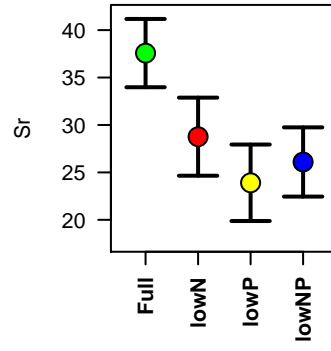

Zn

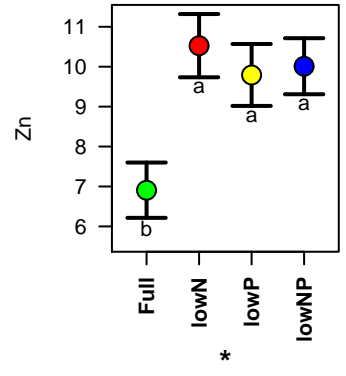

Supplement: Supplementary file 4 — Additional file 4: Figure S4. Ions concentrations for plants grown under Full, LowN, LowP and LowNP. Plots show estimated coefficient and associated standard error. The significance of the treatment effect is shown as *** p < 0.001, ** p < 0.01, * p < 0.05, p < 0.1 (ANOVA; p-value adjusted for multiple tests). Lowercase letters indicate significant (p < 0.05) pairwise differences (Tukey). Figure accompanies MZ66_Ionomics_Analysis in Supplemental File 1. [file 12870_2021_2997_MOESM4_ESM.pdf]

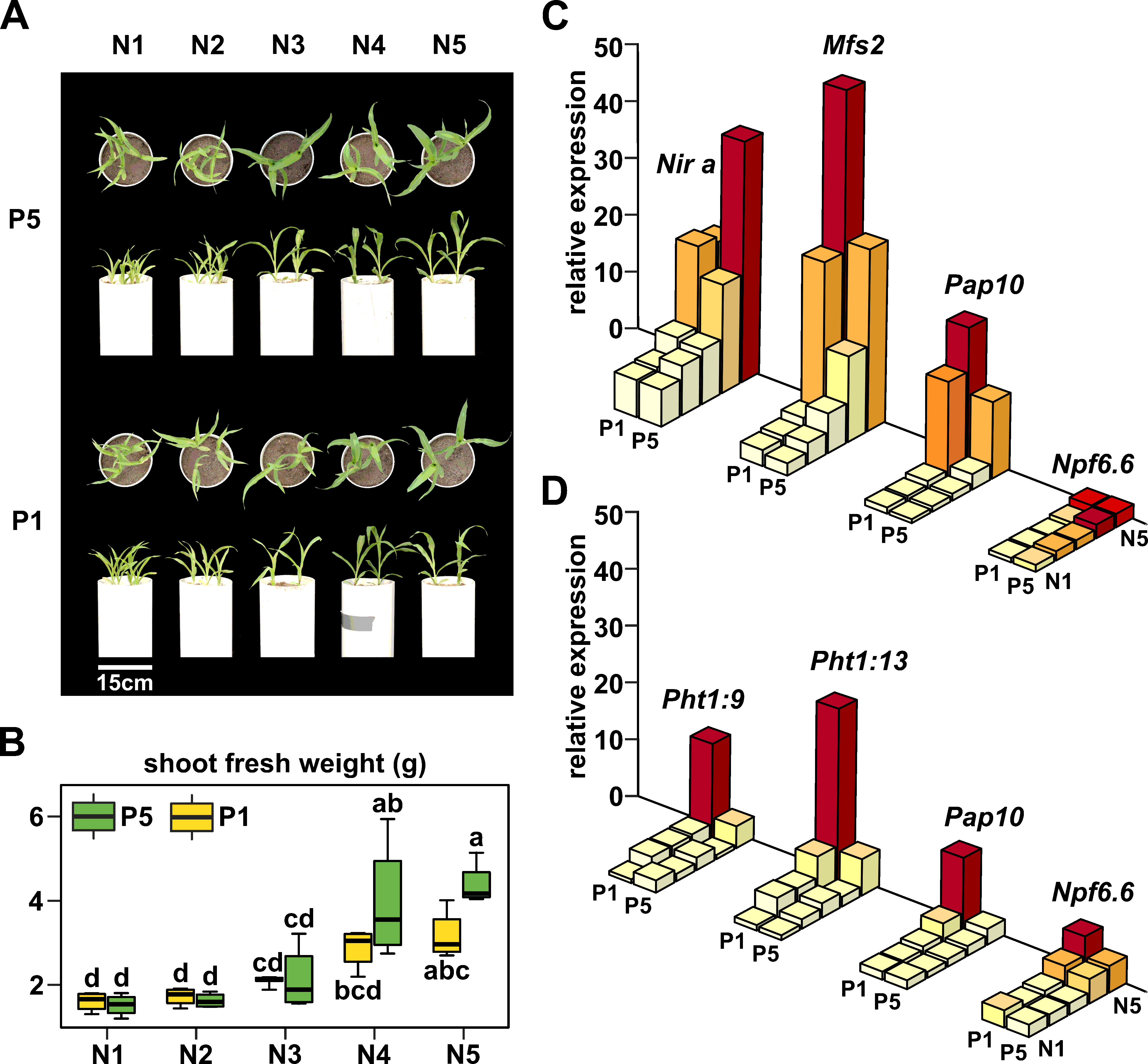

Supplement: Supplementary file 5 — Additional file 5: Figure S5. Transcription of PSR is reduced under LowN availability. Scatter plot showing the distribution of transcript accumulation (log2 fold change, LFC) of 1, 555 genes in A) leaves and B) roots in LowP and lowN. Dotted lines represent LFC of − 1 and 1. Dots filled using heat-colors showing LowNP transcript accumulation. C) Differential transcript accumulation (z, row standardized LFC) with respect to Full of the top 30 (ranked by FDR) classic genes. Figure accompanies MZ67_Selected_Classics in Supplemental File 2. [file 12870_2021_2997_MOESM5_ESM.png]

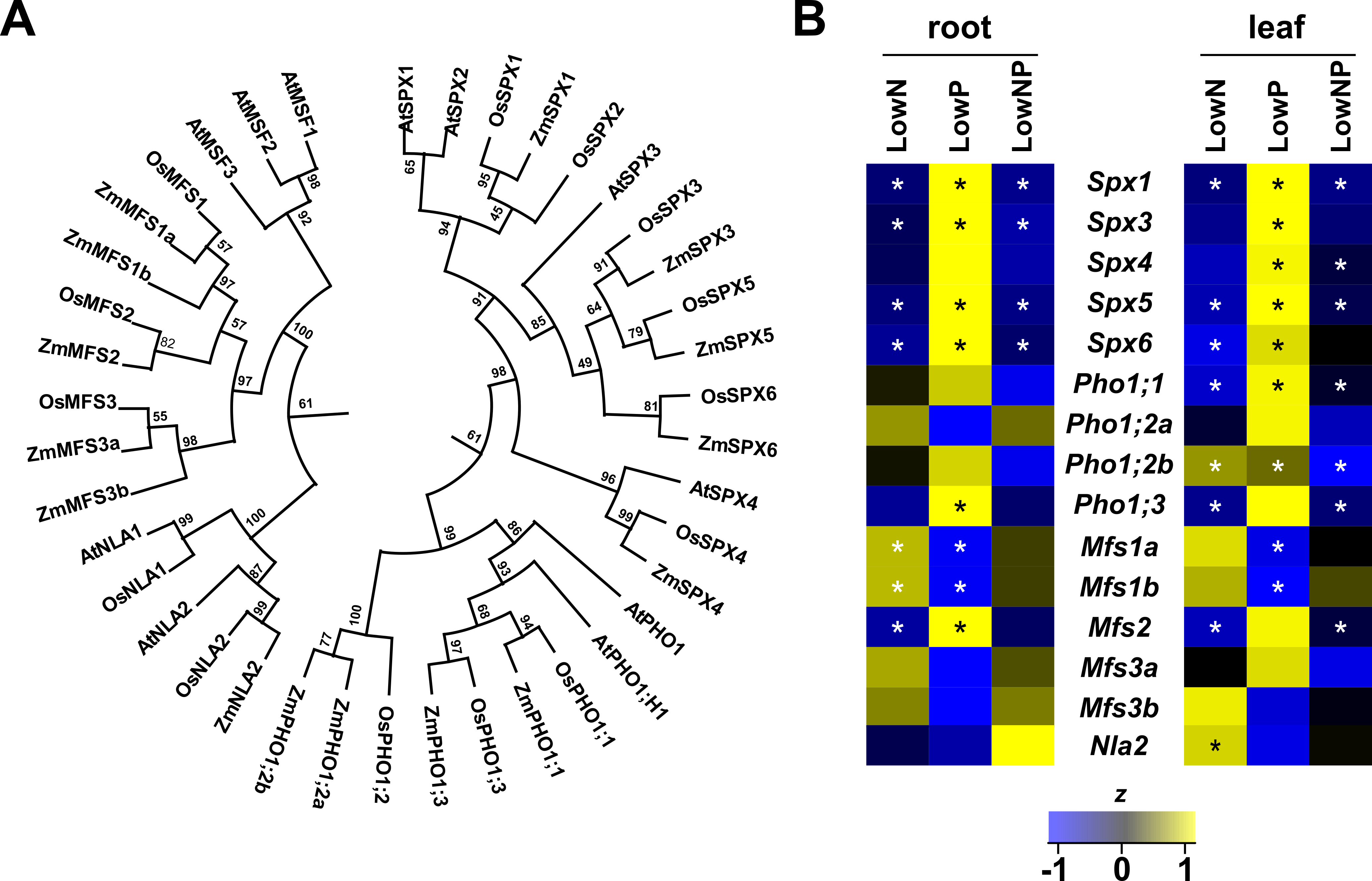

Supplement: Supplementary file 6 — Additional file 6: Figure S6. SPX-domain family members respond to reduced N and P availability. A) Phylogenetic tree of SPX-domain family proteins in maize. Likelihood tree built with Arabidopsis, rice and maize SPX-domain proteins. Numbers at the nodes indicate bootstrap (1000) support as percentage. B) Heat map of maize SPX-domain gene family expression under LowN, LowP and combined LowNP with respect to Full (z, row standardized log2 fold change). Asterisks indicate genes identified as regulated in the transcriptome analysis. [file 12870_2021_2997_MOESM6_ESM.png]
